# Salt-related knowledge, attitudes and practices and their relationship with 24-h urinary sodium and potassium excretions among a group of healthy residents in the UAE: a cross-sectional study

**DOI:** 10.1017/S1368980024002015

**Published:** 2024-10-25

**Authors:** Amjad H Jarrar, Pariyarath S Thondre, Leila Cheikh Ismail, Helen Lightowler, Mo’ath F Bataineh, Alia K Al Baloushi, Amira Y Al Braiki, Shaima Al Halabi, Joudi Hajouz, Usama Souka, Fatima Al Meqbaali, Lily Stojanovska, Habiba I Ali, Johaina T Idriss, Rameez Al Daour, Sheima T Saleh, Maysm N Mohamad, Ayesha S Al Dhaheri

**Affiliations:** 1 Department of Nutrition and Health, College of Medicine and Health Science, United Arab Emirates University, Al Ain, 15551, UAE; 2 Oxford Brookes Centre for Nutrition and Health, Faculty of Health and Life Sciences, Oxford Brookes University, Oxford, UK; 3 Clinical Nutrition and Dietetics Department, College of Health Sciences, University of Sharjah, Sharjah 27272, UAE; 4 Nuffield Department of Women’s & Reproductive Health, University of Oxford, Oxford OX1 2JD, UK; 5 Department of Sport Rehabilitation, Faculty of Physical Education and Sport Science, Hashemite University, Zarqa, Jordon; 6 Institute for Health and Sport, Victoria University, Melbourne, VIC, Australia

**Keywords:** Urinary sodium, Potassium, Food-related knowledge, Sodium sources, UAE

## Abstract

**Objective:**

This study aimed to measure urinary sodium and potassium as a measure of sodium and potassium intake concerning the knowledge, attitude and practice towards sodium intake among a group of healthy residents in the UAE.

**Design:**

A cross-sectional study on a sample of healthy adults in the UAE. In addition to the knowledge, attitude and practice questionnaire, sodium and potassium excretions and food records were taken.

**Setting:**

The UAE.

**Participants:**

A sample of 190 healthy individuals aged between 20 and 60 years.

**Results:**

The mean (± s d) age of the sample was 38·6 (± 12·5) years, and 50·5 % were females. The mean urinary sodium and potassium intake were 2816·2 ± 675·7 mg/d and 2533·3 ± 615 mg/d, respectively. The means were significantly different compared with the WHO recommendation of sodium and potassium (*P* < 0·001). About 65 % of the participants exceeded the WHO recommendations for salt intake, and participants’ knowledge classification for health-related issues was fair, while food-related knowledge was poor (*P* = 0·001). A two-stage stepwise multiple regression analysis revealed that knowledge, attitude and practice scores were negatively associated with urinary sodium excretion (*r* = –0·174; *P* = 0·017) and those older participants and females had lower urinary sodium excretion (*P* < 0·001).

**Conclusions:**

These findings may suggest an increase in the risk of hypertension in the UAE population. Moreover, these findings emphasise the need to establish education and public awareness programmes focusing on identifying the sodium contents of foods and establishing national regulations regarding food reformulation, particularly for staple foods such as bread.

Non-communicable diseases (NCD), also known as chronic diseases, tend to be of long duration and are the result of a combination of genetic, physiological, environmental and behavioural factors. The major burden of NCD is accounted for by diabetes mellitus, CVD, cancers and chronic respiratory diseases (such as chronic obstructive pulmonary disease and asthma). These diseases are steadily increasing globally, as documented by the WHO, and are considered as leading causes of death worldwide^(1)^. NCD contribute to the death of forty-one million people each year, equivalent to 71 % of all deaths globally^(2)^. CVD account for most NCD deaths with 17·9 million annually, followed by cancer (9·3 million), respiratory diseases (4·1 million) and diabetes (1·5 million). Collectively, these four groups of diseases account for over 80 % of all premature NCD deaths^(2)^.

In developing countries, CVD are causes of great concern as it is most commonly attributable to risk factors such as obesity, high blood pressure (BP), lack of physical activity and smoking^(3)^. Hypertension, which is associated with increased BP, is a major risk factor in the development of CVD, which are responsible for 62 % of strokes and 49 % of CHD worldwide^(3)^. Hypertension is a common medical condition; its prevalence increases with age and is estimated to affect 65 % of those ≥ 60 years old^(4)^. An estimated 20 % of the global population will be ≥ 65 years old by 2023^(5)^. Therefore, the impact of high BP on mortality among older adults is expected to grow over the coming decades. According to the WHO, CVD account for 46 % of the total deaths in the Kingdom of Saudi Arabia, 41 % in Kuwait, 33 % in the Sultanate of Oman, 30 % in the UAE, 26 % in Bahrain and 24 % in Qatar^(6)^.

Salt is an ionic compound of sodium and chloride. Sodium is an essential nutrient necessary for the maintenance of certain body functions, such as keeping fluid balance and maintaining muscle and nerve functions. According to the WHO, adults should consume less than 5 g (just under a teaspoon) of salt per d^(4)^. In the Eastern Mediterranean region, the current salt intake is high, with an average intake of > 12 g per person per d^(6)^. These intakes are concerning since high sodium (> 2 g/d or > 5 g salt/d) and insufficient potassium intake (< 3·5 g/d) can contribute to high blood pressure and increase the risk of heart disease and stroke^(2)^.

Research has shown that a decrease in salt intake to 6 g predicted a fall in blood pressure of ∼7/4 mmHg in hypertensive and of ∼3·5/2 mmHg in normotensive individuals^(7)^. This decrease in blood pressure is predicted to lead to a 14 % reduction in stroke deaths and a 9 % reduction in coronary artery disease deaths in hypertensive people, as well as a 6 % reduction in stroke deaths and 4% reduction in coronary artery disease deaths in normotensive people^(7)^. Population salt reduction is considered a ‘best buy’ intervention for the prevention of NCD^(7)^. CVD in adults are associated with hypertension, and high sodium intake is a leading cause of hypertension. Conversely, potassium attenuates sodium’s negative effects. Increased potassium intake is associated with reduced systolic blood pressure and the risk of developing CVD^(8)^. The WHO has suggested a sodium-to-potassium ratio of approximately 1·00, which will be associated with a low risk for the development of CVD^(3)^. The WHO has recommended different strategies to reduce salt intake at country levels. These include consumer education and awareness, food reformulation, front-of-pack labelling and salt taxation^(9)^. Although the specific strategies vary in approach, some strategies use a multi-component approach. However, consumer education is the most common intervention; therefore, assessing consumer knowledge, attitude, and practice (KAP) is the recommended approach to set appropriate consumer education programmes^(10)^.

The WHO recommends using a 24-h urinary sodium excretion as the gold standard method to estimate sodium intake as a more practical method in comparison with dietary surveys^(11,12)^. To date, only one study has used 24-h urinary sodium excretion as a tool to assess salt intake in UAE in 2015/2016^(10)^. Therefore, this study aimed to measure urinary sodium and potassium excretions according to the Pan American Health Organization (PAHO)/WHO protocol^(12)^, to predict sodium and potassium intakes and to measure the ratio of sodium-to-potassium intake. Additionally, it aimed to assess the relationship between urinary sodium and potassium excretions and KAP regarding salt intake.

## Materials and methods

### Study design

A cross-sectional study was conducted between October 2018 and March 2019 in the UAE. Several methods were used for patient recruitment: email circulation to students, staff and faculty members of UAE University (Al Ain), University of Sharjah (Sharjah) and Zayed University (Abu Dhabi). In addition, posters were displayed (in shopping malls, health centres, hostels and in the country) and on social media including Snapchat™ and WhatsApp™.

**Figure 1.**
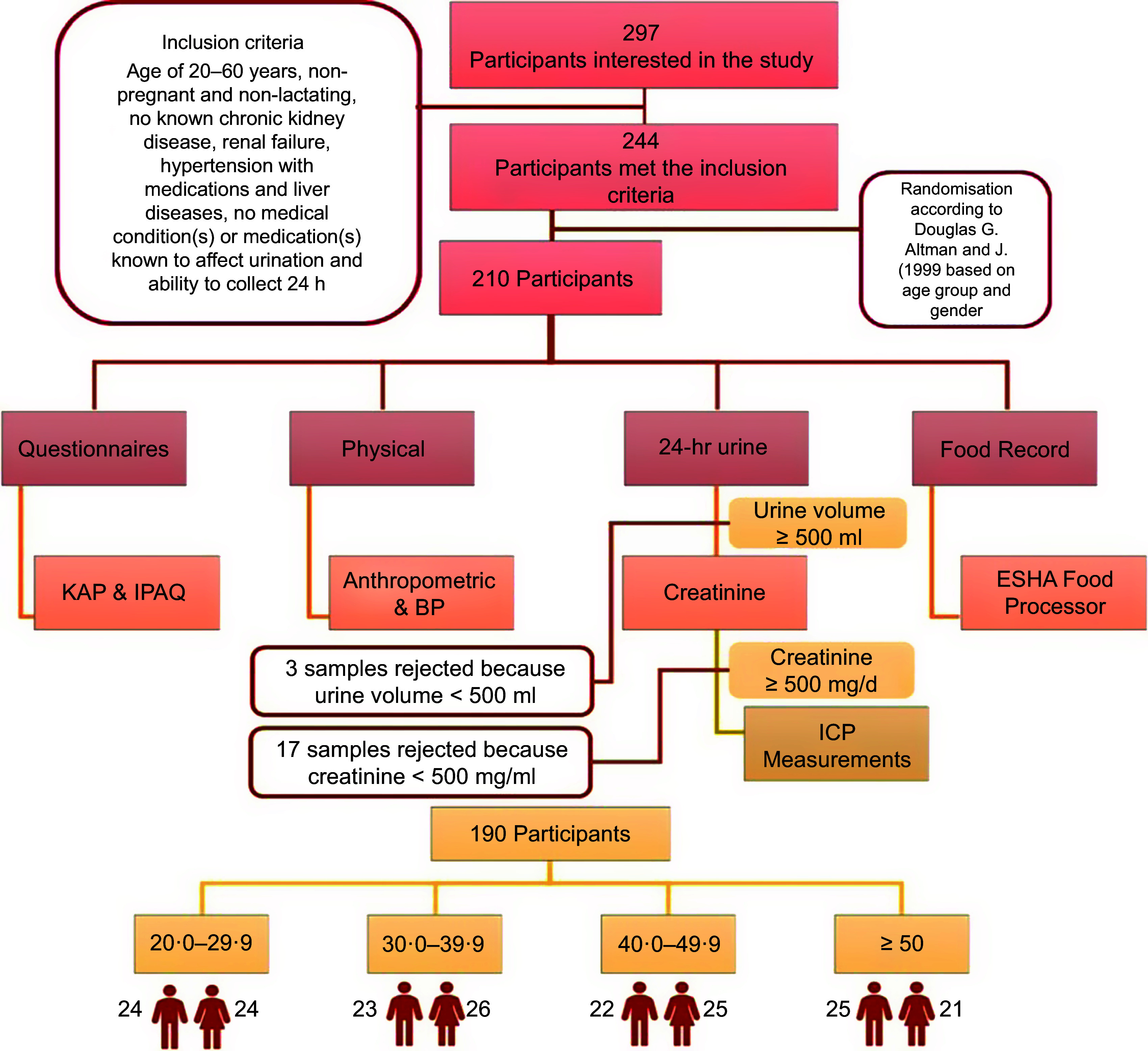
Flow chart of the study. KAP, knowledge, attitude and practice; IPAQ, International Physical Activity Questionnaire; ICP, inductively coupled plasma.

A sample of 297 healthy individuals aged between 20 and 60 years showed interest in participating in the study from the seven Emirates: the Western region (Emirate of Abu Dhabi, including Abu Dhabi and Al-Dhafra districts), Northern region (Dubai, Sharjah, Ajman, Ras Al Khaimah, Fujairah and Umm Al Quwain) and Eastern region (Al Ain). Only 244 met the following inclusion criteria at screening as self-report, including the age of 20–60 years (male and female), non-pregnant and non-lactating, no known chronic kidney disease, renal failure, hypertension with medications and liver diseases, no medical condition(s) or medication(s) known to affect urination and ability to collect 24-h urine (Fig. 1)^(11)^. Randomisation for the sample was performed using Altman and Bland procedure^(13)^, according to gender and four age groups. According to age groups and gender, 210 out of 244 (86·1 %) healthy participants were selected to participate in this study. Participants were divided into four age groups: 20–29, 30–39, 40–49 and 50 and above. All participants provided written informed consent to participate in the study.

The sample size was determined based on the WHO Eastern Mediterranean Region Office protocol, published in 2010 for 24-h urine collection and analysis^(12)^. To be able to detect a minimum difference in sodium excretion with 20–35 mmol, which is equivalent to approximately 500 mg with a sd of 20–30 mmol, the required sample size for each age group per gender was around 21–25 participants. Therefore, the recommended sample size for each age group with both genders with a ratio of 1:1 was 46 with four age groups with a total number of 188 (alpha = 0·05, power = 0·83).

The exclusion criteria for this study were implemented at two stages: at the screening (self-report) and after urine collection; those who were unable to collect adequate urine within 24 h (i.e. volume < 500 ml) or whose urine samples had creatinine level reference range of 500–2000 mg/d, which is equivalent to 9–26 mg/kg of body mass for female participants and 13–29 mg/kg of body mass for male participants, were excluded^(10,14)^.

This study was approved by the University of Sharjah Research Ethics Committee (blinded for peer review) and was conducted according to the stated principles in the Declaration of Helsinki. All participants were asked to read and sign a written informed consent form.

### Anthropometric measurements

Body weight and height were measured for each participant, and their BMI was calculated as weight (kg) divided by height (m) squared (kg/m^2^). Height was recorded to the nearest 1 cm using a stadiometer (Seca Stadiometer, Seca Ltd), and weight was recorded using a balance (Seca Stadiometer, Seca Ltd) to the nearest 0·1 kg^(15)^. Systolic and diastolic blood pressures were measured in triplicate after 5 min of resting in a seated position using a calibrated digital automated blood pressure monitor (Omron HEM-907-E7). The measurements were taken at 3-min intervals, and the average of the last two readings was used. Participants were designated as having normal blood pressure if their systolic BP was < 120 mmHg and/or diastolic BP was equal to < 80 mmHg^(16)^.

### Assessment of food intake and physical activity

The aim of collecting food records on the same day as the 24-h urine collection is to estimate participants’ protein intake and compare it with 24-h urinary creatinine excretion, as well as to ensure the adequacy of a 24-h urine sample. Participants were asked to record their food intake on the same day as the urine collection using a 24-h record. In the food records, all consumed food and beverages (excluding leftovers) were documented with detailed information, including brand names, preparation methods (e.g. adding salt, sugar, oil, butter or ghee), measurements (e.g. teaspoon or tablespoon) and cooking methods (e.g. deep frying, grilling or boiling). This approach was consistent with the methods reported in sources^(15)^ and used in studies^(17,18)^. In addition to recording the quantities of food intake, participants were asked to record the use and the quantities of chicken cubes, monosodium glutamate or any other sauces and dressing oils. All participants were given verbal and provided with written instructions on how to determine food portion size. Food photographs with different portion sizes were used to aid participants in estimating the correct portion size consumed. The food record was collected by the research team at the same time as collecting urine bottles. The Food Processor® Nutrition and Fitness Software–ESHA food analysis programme (version 10.4) and Kuwaiti Food Composition database were used to analyse the energy and macronutrient contents of foods (carbohydrate, protein and fat)^(19)^. ESHA software was chosen for the analysis of food intake by several studies conducted in the UAE^(17,18)^.

Physical activity for the participants was measured using a 7-d self-reported short International Physical Activity Questionnaire, associated with scoring points based on time spent per d and week according to the type of activity levels^(20)^. Physical activity classification was performed according to Forde (2018)^(21)^ into three categories (low, moderate and high) levels of physical activity.

### Knowledge, attitude and practice questionnaire (KAP)

Participants were asked to complete a self-reported questionnaire. The questionnaire assessed the knowledge related to salt and health outcomes, frequency of consumption and perceived salt consumption and was developed according to the WHO/PAHO^(12)^ recommendations for the assessment of population sodium intake and behaviours. In addition, this questionnaire was used by other previous studies conducted in the UAE to measure KAP^(10,22)^. The KAP questionnaire consists of forty-four items and is divided into three components: knowledge, attitude and practice. The knowledge component includes twenty-eight questions, and it is divided into food and health-related knowledge (nineteen and nine items, respectively). Food-related knowledge questions included nineteen questions, the questionnaire inquired about the percentage of sodium in salt (response options: ‘20 %’, ‘40 %’, ‘60 %’ and ‘I don’t know’) and other food-related questions were to classify specific foods as high or low in salt/sodium; these food items included Arabic bread, Iranian bread, Cheddar cheese, rice, processed meat, chicken cubes, pickles, salad dressings, instant noodles, fresh vegetables and canned vegetables^(10,22)^. Each correct response earned 1, and the incorrect response earned 0^(10)^. The total score for food-related knowledge of nineteen items was converted into a score out of 10 by dividing the obtained score over 19 and multiplying it by 10. The health-related knowledge questionnaire included questions about the relationship between high salt intake and specific health conditions such as hypertension, CVD, fluid retention, fever, renal diseases and diabetes and if participants think that reducing salt intake improves their blood pressure and overall health (response options: ‘yes’, ‘no’, ‘I don’t know’). Similarly, health-related knowledge was converted from a total score of 9 into a score of 10 by dividing the obtained score over 9 and multiplying it by 10.

The attitude and practice component includes sixteen questions. Attitude components include five questions related to how much salt you consume, whether are you concerned about the amount of salt consumed and whether reducing salt intake is important to you. The practice component of the questionnaire includes eleven questions, and it contains questions about the use of food labels in purchase and the frequency of adding salt during food preparation, cooking or at the table. Attitudes and practice were assessed by a three-point Likert scale, with answers of ‘Agree or Often or Yes’ or sometimes with ‘Disagree not often or No’. Agree or yes was associated with a positive attitude or practice, sometimes with a neutral attitude or practice and no or not often with a negative attitude and practice^(10)^. The development and performance of the specific questionnaire have been described elsewhere^(10)^.

### 24-h urine collection and analysis

A single 24-h urine collection was obtained for the estimation of sodium excretion. Participants were given written and verbal instructions for the 24-h urine collection procedure. A 3-litre coded plastic bottle was given to each participant for urine collection. Participants were asked to discard the first urine of the day and to collect all the urine in the plastic bottle provided over the following 24 h. Participants were also asked to write on a separate sheet the time and date at the start and end of the urine collection, indicating occasions they missed collecting urination. The 3-litre coded plastic bottle was collected by the research team after a 24-h completing the test. Urine analysis for sodium, potassium and creatinine levels was conducted in the College of Agriculture and Veterinary Medicine laboratories at the United Arab Emirates University. For the measurement of sodium and potassium levels in the urine, 50 ml of the urine sample was mixed with 200 µl of 1 % nitric acid. Analytical solutions were introduced to a Varian ICP-OES model 710-ES spectrometer for sodium and potassium measurements^(23)^. Salt was calculated from urinary sodium excretion (24 h) multiplied by a conversion factor of 2·5^(14)^. Potassium intake was calculated from urinary potassium excretion (24 h) × conversion factor of 1·3^(24)^, and 24-h urinary creatinine was measured using the ab204537 Creatinine Assay Kit based on colorimetric assay^(25)^ with a UV-visible spectrophotometer (Multiskan Go, Thermo-Fisher Scientific). The assay relies on the Jaffe’ reaction^(26)^.

### Statistical analysis

Data were recorded and analysed using the Statistical Package for Social Sciences (SPSS) software, version 25 (SPSS). The normality of data across a combination of independent variables was tested using the Shapiro–Wilk test. Continuous variables were presented as mean ± sd, and categorical variables were expressed as numbers and percentages. Descriptive statistics were used to summarise the baseline characteristics of the study participants. A two-way ANOVA test was performed to assess the main effects of gender and age groups on 24-h urinary sodium, potassium and creatinine excretions.

For interaction analysis, one-way ANOVA, followed by Bonferroni *post hoc* test, and independent *t* test were used to compare dependent variables according to gender and age groups. One-sample *t* test was used to compare the mean urinary sodium, salt and potassium excretions with the RDA, and an assessment was carried out based on gender. The effect size was calculated as Cohen’s *d* for the *t* test and partial eta-squared (ηp2) for ANOVA.

A *χ* ^2^ test was used for the categorical variables. Univariate analysis was carried out to select covariates for the final model. Only variables with a *P*-value less than 0·2 were included in the final two-stage hierarchical multiple regression. A two-stage hierarchical multiple regression was performed to assess the association of urinary sodium and potassium excretions (dependent variables) with KAP after controlling for the influence of age, gender, marital status and energy intake. Statistical significance was set as a *P*-value < 0·05.

## Results

### Sample characteristics

A total of 210 participants provided urine specimens out of which three participants were excluded due to limited urine sample collection (< 500 ml urine), and a further seventeen participants were excluded due to creatinine levels below 500 mg/d in the urine. A final sample size of 190 participants (alpha = 0·05, power = 0·83) was included in the analysis as shown in Fig. 1. Table 1 shows the socio-demographic and physical characteristics of the study participants. The mean age of the participants was 38·6 ± 12·5 years, with 50·5 % females. The mean BMI for participants was 25·4 ± 4·8 kg/m². About 50 % of the study population had normal blood pressure. The prevalence of overweight and obese individuals was 32·8 % and 14·3 %, respectively. Only 2·1 % of the participants had a high level of physical activity.

**Table 1.** Socio-demographic and physical characteristics of study participants (*n* 190)

Characteristics	*n*	%
Age (years)	190	100
20–29·9	48	25·3
30–39·9	49	25·8
40–49·9	47	24·7
≥ 50	46	24·2
Gender		
Female	96	50·5
Male	94	49·5
Nationality		
Emirati	78	41·1
Arabs	112	58·9
Geographic distribution by region		
Northern region	60	31·6
Eastern region	68	35·8
Western region	62	32·6
Educational level		
Undergraduate university level	166	87·4
School level (intermediate or lower)	13	6·8
Postgraduate level	11	5·9
Employment status		
Student	81	42·6
Unemployed	30	16·0
Employed	79	41·4
Marital status		
Single	67	35·3
Married	116	61·1
Divorced	7	3·7
BMI (kg/m²)		
Underweight (18·5)	14	7·4
Normal weight (18·5–24·9)	87	45·8
Overweight (25–29·9)	62	32·6
Obese	27	14·2
Physical activity levels^b^		
Low level of physical activity^c^	89	46·8
Moderate level of physical activity^c^	97	51·1
High level of physical activity^c^	4	2·1
Blood pressure (mmHg)		
Normal (< 120/80)	100	52·6
Elevated (systolic 120–129 and diastolic < 80)	46	24·2
High blood pressure stage 1 (systolic 130–139 or diastolic 80–89)	44	23·2

^a^Data are presented as mean (sd) for continuous variables or number (%) for categorical variables. ^b^According to International Physical Activity Questionnaire (IPAQ)^(23)^. ^c^Forde C.^(24)^.

### Mean 24-h urinary sodium, potassium and creatinine

Table 2 shows the urinary (24 h) sodium, potassium and creatinine excretions among the study participants. The mean sodium excretion in urine was 2816·2 ± 675·7 mg/d, which is equivalent to 7040 ± 1689·3 mg/d of salt. The mean value for sodium excretion in urine exceeded the WHO recommendations and the RDA for sodium intake of less than 2300 mg. Of the 190 participants, 131 (65·3 %) had a sodium excretion above the WHO-recommended level of 2300 mg. A significant difference was observed in mean urinary sodium excretion for male participants compared with female participants (*P* = 0·001). The mean potassium intake was lower than the WHO recommendations of 3510 mg/d. There was a significant difference in the potassium intake between male and female participants (*P* = 0·007). The mean 24-h urinary creatinine excretion for females and males was within the normal range of 24-h creatinine excretion as mg/d and as mg/kg per d^(14)^.

**Table 2.** Urinary (24 h) sodium, potassium and creatinine excretions (*n* 190)

Variables	Mean (sd) ^a^						*P*-value^c^
	Total		Female (*n* 96)		Male (*n* 94)		
	Mean	sd	Mean	sd	Mean	sd	
Urinary sodium (mg)	2816·2	675·7^b^	2671·7	545·3^b^	2963·7	761·9^b^	0·003
Salt (mg)	7040	1689·3	6679·3	1363·3	7409·3	1904·9	0·003
Urinary potassium (mg)	1948·7	473·1	1857·5	440·8	2041·8	488·9	0·007
Potassium intake (mg)	2533·3	615^d^	2414·7	573^d^	2654·4	635^d^	0·007
Sodium-to-potassium intake ratios	1·15	0·30	1·15	0·29	1·16	0·32	0·872
Creatinine 24 h (mg)	1440·5	325·0	1223·9	225·8	1621·7	254·6	0·001
Creatinine (mg/kg per 24 h)	18·1	3·2	16·1	2·6	20·6	3·5	0·001

^a^Data presented as mean ± sd. ^b^Significantly higher than the WHO recommendations for sodium (one-sample *t* test used to compare mean with RDA = 2300 mg/d, significance at *P* < 0.05). ^c^Independent-samples *t* test used to assess significance according to gender (*P* < 0.05). ^d^Significantly lower than the WHO recommendations for potassium (one-sample *t* test used to compare mean with RDA = 3510 mg/d, significance at *P* < 0.05).

**Figure 2.**
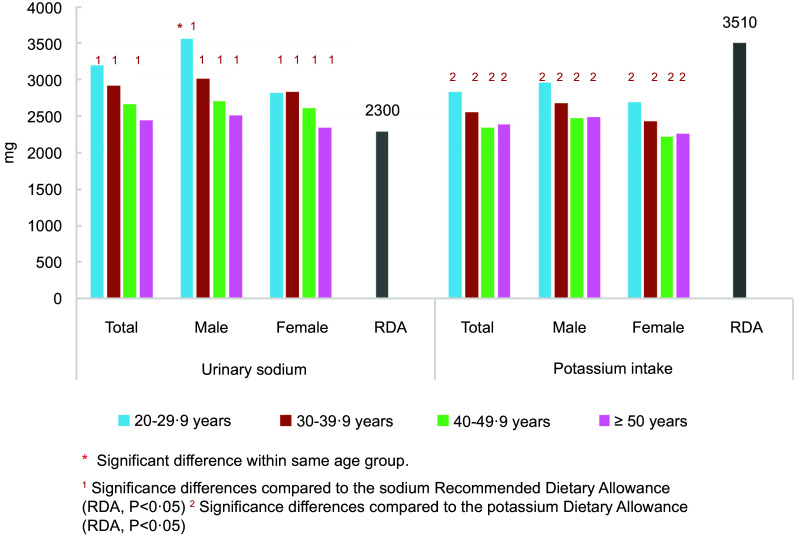
Urinary sodium and potassium excretions according to gender and age groups.

Figure 2 illustrates the urinary sodium and potassium excretions according to gender and age groups. The maximum urinary sodium excretion was detected among male participants in the age group of 20–29·9 years. Moreover, the maximum urinary sodium excretion was detected in female participants in the age group of 30–39·9 years. All age groups showed a significant difference in the intake of sodium and potassium compared with the RDA.

### Salt-related knowledge, attitudes and practices

Table 3 presents the mean food-related and health-related knowledge scores. Female participants had significantly higher scores for food-related knowledge compared with male participants (*P* = 0·008). However, both were classified as poor food-related knowledge (below 6 out of 10)^(22)^. Health-related knowledge for each gender was classified as fair (between 6 and 7 out of 10)^(22)^. This suggests that health-related knowledge was significantly higher compared with food-related knowledge (*P* < 0·05).

**Table 3.** Food and health-related knowledge scores for the study population

Variables	Mean (sd) ^a^									*P*-value ^d^
	Total (*n* 190)			Female (*n* 96)			Male (*n* 94)			
	Mean	sd	C^e^	Mean	sd	C^e^	Mean	sd	C^e^	
Foods-related knowledge^b^	5·1	2·3	Poor	5·5	2·0	Poor	4·6	2·5	Poor	0·008
Health-related knowledge^b^	6·2	2·9	Fair	6·4	2·9	Fair	6·1	2·9	Fair	0·562
*P*-value^c^	0·001			0·009			0·001			

^a^Mean (sd). ^b^Knowledge scores are reported out of 10. ^c^Paired sample *t* test used to compare means for health and food-related knowledge (*P* < 0·05). ^d^Independent-samples *t* test used to assess significance at *P* < 0·05 for variables according to gender. ^e^Classification according to the score for knowledge < 6 classified as ‘Poor’, 6–7 classified as ‘Fair’, > 7 classified as ‘Good’^(25)^.

**Figure 3.**
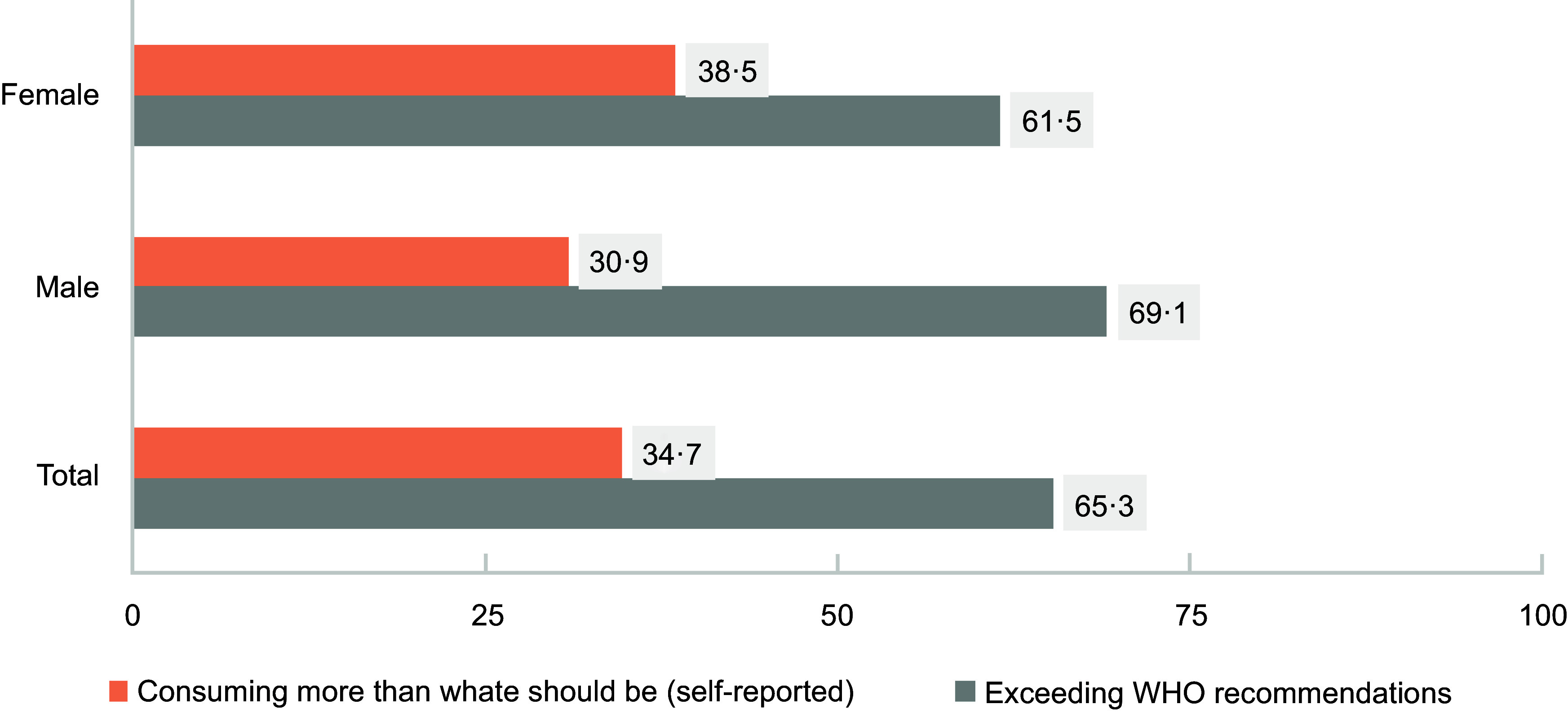
Percentage of the study population with actual urinary sodium excretion exceeding WHO recommendations compared with the percentage of participants reporting excess sodium intake.

As shown in Fig. 3, almost 65 % of the study population exceeded the WHO recommendation for sodium intake (69·1 % of male participants and 61·5 % of female participants).

Table 4 illustrates that only 20 % of the participants reported ‘too much salt intake’ and only 21 % had concerns about the amount of salt/sodium in their diet. Moreover, only 15·8 % of the study population tended to check food labels often, with a higher percentage of female participants than males (*P* = 0·008). Out of these, only 8 % reported checking the sodium contents of the food often. Most of the study population (60 %) reported that they often add salt during cooking. Almost 19 % of male participants reported adding salt before tasting the food, which was significantly higher than females (*P* = 0·001). Almost 45 % of the study population reported having tried to reduce salt intake before.

**Table 4.** Salt-related attitudes and practice among the study population by gender (*n* 190)

	Total^b^		Female		Male		*χ* ^2^ ^c^	*P*-value
Variable^a^	*n*	%	*n*	%	*n*	%		
Attitudes among the study population								
How much salt do you think you consume (Just the right amount)	134	70·5	69	71·9	65	69·1	0·742	*P* = 0·690
Are you concerned about the amount of salt/sodium in the diet (Yes)	21	11·1	9	9·4	12	12·8	1·534	*P* = 0·464
Reducing added salt to foods is important to you (Agree)	49	25·8	26	27·1	23	24·5	4·114	*P* = 0·128
Reducing consumption of processed foods is important to you (Agree)	80	42·1	35	36·5	45	47·9	3·483	*P* = 0·175
Reducing your sodium intake is important to you (Agree)	39	20·5	14	14·6	25	26·6	6·115	*P* = 0·047
Practice among the study population								
Check food labels (Often)	30	15·8	23	24·0	7	7·5	9·742	0·008
Information on food labels affects purchasing decisions (Often)	17	9·0	10	10·4	94	7·4	0·660	0·719
Check labels specifically for salt/sodium content (Often)	15	8·0	7	7·3	8	8·5	1·723	0·423
Salt/sodium content on label affects purchasing decisions (Often)	13	6·8	7	7·3	6	6·4	2·552	0·279
Try to buy ‘low-salt’ foods (Often)	25	13·2	11	11·5	14	14·9	0·495	0·781
Add salt to food during cooking (Often)	120	63·2	62	64·6	58	61·7	0·661	0·719
Use stock cubes during cooking (Often)	33	17·4	19	19·8	14	14·9	0·795	0·672
Add salt to food at the table (Often)	24	12·6	11	11·5	13	13·8	1440·0	0·487
Add salt before tasting the food (Often)	23	12·1	5	5·2	18	19·2	13·681	0·001
Did you try to reduce salt intake before (Yes)	89	46·8	46	47·9	43	45·7	0·657	0·720
Did you try to use spices (lemons and herbs) to reduce salt (Yes)	65	34·2	37	38·5	28	29·8	4·266	0·118

^a^Practice was assessed based on a three-point Likert scale but only answers of ‘Agree or Often or Yes’ are presented. ^b^Data presented as number and frequencies of agree or yes answers. ^c^ *χ* ^2^ test used to compare variable association with gender (*P* < 0·05).

Table 5 shows that most of the study sample failed to identify Arabic/Iranian bread and cornflakes as sources of high salt/sodium. On the other hand, most of the study samples (60–75 %) were able to identify instant noodles, chicken stock cubes, cheddar cheese, salad dressing, tomato paste, canned vegetable foods and pickles as sources of high salt/sodium. Moreover, female participants had significantly higher scores in identifying foods with high salt contents, such as cheddar cheese, ketchup, tomato paste and cornflakes, and low-sodium contents, such as basmati rice, milk and yogurt, compared with males (*P* < 0·05).

**Table 5.** The number and percentage of participants who answered correctly on food-related knowledge

	Total		Female		Male		
Sodium contents of selected foods^a^	*n*	%	*n*	%	*n*	%	*P*-value^c^
Arabic bread (high)	66	34·7^b^	35	36·5	31	33·0	0·219
Iranian bread (high)	68	35·8	38	39·6	30	31·9	0·302
Rice-Egyptian (low)	100	52·6	54	56·3	46	49·0	0·107
Rice-Basmati (low)	106	55·8	60	62·5	46	49·0	0·018
Milk/yogurt (low)	100	52·6	56	58·3	44	46·8	0·07
Fresh red meat (low)	66	34·7	29	30·2	37	39·4	0·721
Fresh poultry (low)	95	50·0	43	44·8	52	55·3	0·533
Fruits (low)	132	69·5	70	72·9	62	66·0	0·182
Fresh vegetables (low)	135	71·1	69	71·9	66	70·2	0·622
Canned vegetables (high)	119	62·6	67	69·8	52	55·3	0·058
Cheddar cheese (high)	137	72·1	77	80·2	60	63·8	0·016
Pickles (high)	141	74·2	74	77·1	67	71·3	0·484
Olive oil (low)	101	53·2	48	50·0	53	56·4	0·271
Salad dressing oil (high)	126	66·3	69	71·9	57	60·6	0·198
Ketchup (high)	113	59·5	64	67·3	49	0·52	0·032
Tomato paste (high)	123	64·7	69	71·9	54	57·4	0·009
Cornflakes (high)	50	26·3	32	33·3	18	19·1	0·015
Chicken cubes (high)	115	60·5	63	65·6	52	55·3	0·083
Instant noodle (high)	110	58	60	62·5	50	53·2	0·087

^a^The correct answers are provided in brackets next to each variable. ^b^Data presented as number and frequencies of the correct answer. ^c^Independent-samples *t* test used to assess significance at *P* < 0·05 for variables according to gender.

### Dietary intake and physical activity

Table 6 presents macronutrient intake among the study sample (*n* 190), with a mean ± se for energy, carbohydrates, protein and fat intakes of 1850·8 ± 33·8 kcal, 232·0 ± 6·4 g, 74·5 ± 1·1 g and 69·3 ± 1·8 g, respectively. The percentage of energy from carbohydrates, protein and fat was within the Acceptable Macronutrient Distribution Range for the study population within 45–65 % for carbohydrates, 10–35 % for protein and 25–35 % for fat.

**Table 6.** Daily energy and macronutrient distribution for the study population

	Mean (se)¹									
	Total			Females (*n* 96)			Male (*n* 94)			*P*-value ^3^
Variables	Mean	se	%	Mean	se	%	Mean	se	%	
Energy (kcal)	1850·8	33·8		1816·0	38·5		1918·2	55		0·833
Carbohydrate²	232·0	6·4	50·2 %	228·2	8·5	50·3 %	238·2	9·6	49·6 %	0·851
Protein (g) (%)	74·5	1·1	16·1 %	70·9	1·1	15·6 %	79·7	1·9	16·6 %	0·667
Fat (g) (%)	69·3	1·8	33·7 %	67·0	2·6	33·2 %	71·9	2·5	33·8 %	0·633
Cholesterol (mg)	181·8	9·2		200·2	8·0		215	16·0		0·126
Fibre (g)	15·1	0·9		13·9	1·1		16·4	1·4		0·181

¹Data presented as mean ± se. ²Data displayed in grams and % from total calorie. ^3^Independent-samples *t* test used to assess significance at *P* < 0·05 for variables according to gender.

### Predictors of urinary sodium and potassium excretions

Table 7 illustrates the association between KAP total scores with urinary sodium and potassium excretion. A two-stage stepwise multiple regression analysis revealed a negative relationship between KAP and urinary sodium excretion. In the first model (step 1, Table 7-A), four variables including age, gender, marital status and energy intake were entered. Age and gender were good (significant) predictors of urinary sodium excretion (*P* < 0·05); however, marital status (*P* = 0·175) and energy intake (*P* = 0·672) were not significantly associated with urinary sodium excretion. The four variables in step 1 explained 22·4 % (F (4, 185) = 13·356, *P* < 0·001; R² = 0·224, *P* < 0·001) of the variance in urinary sodium excretion. In step 2, the KAP total variable was entered while controlling for age, gender, marital status and energy intake. Adding the KAP total variable significantly explained an additional 2·4 % of the urinary sodium excretion variance (R² change = 0·024, *P* = 0·017). Overall, the five variables explained 24·8 % (F (5, 184) = 12·113, *P* < 0·001) of the variance in the urinary sodium excretion. In step 2, when all of the five variables were in the final model, two variables failed to predict urinary sodium excretion, marital status (*P* = 0·104) and energy intake (*P* = 0·844), while age (B = –25·040, *P* < 0·001, *r* = –0·435), gender (males) (B = 349·094, *P* < 0·001, *r* = 0·281) and KAP (B = –6·840, *P* < 0·017, *r* = –0·174) were predictors for urinary sodium excretions. These results showed a negative correlation between KAP and urinary sodium excretion. Furthermore, age was inversely associated with urinary sodium excretion. Males were more likely to show higher urinary sodium excretions than females.

**Table 7.** Stepwise multiple regression to assess predictors of urinary sodium and potassium excretions

Model	A- Urinary sodium excretion¹					
	Variables^2^ (unit)	B^3^	CI 95 %	Se ^4^	R	*P*-value
1	Age (> 30 years)	−24·245	–31·848, –16·642	3·854	−0·420	< 0·001
	Gender (males)	317·957	143·946, 491·968	88·202	0·256	< 0·001
	Marital status (married)	97·838	–43·848, 239·523	71·817	0·100	0·175
	Energy intake (kcal)	0·035	–0·129, 0·199	0·083	0·031	0·672
2	Age (> 30 years)	−25·040	–32·576, 17·504	3·820	−0·435	< 0·001
	Gender (males)	349·094	175·381, 522·807	88·048	0·281	< 0·001
	Marital status (married)	116·679	–24·075, 257·433	71·342	0·120	0·104
	Energy intake (kcal)	0·016	–0·146, 0·179	0·082	0·015	0·844
	KAP total^5^	−6·840	–12·461, –1·220	2·849	−0·174	0·017
	B- Urinary potassium excretion^6^					
1	Age (> 30 years)	−12·303	–17·930, –6·677	2·852	−0·302	< 0·001
	Gender (males)	196·585	67·820, 325·349	65·268	0·216	0·003
	Marital status (married)	101·193	–3·651, 206·037	53·143	0·139	0·058
	Energy intake (kcal)	0·061	–0·060, 0·183	0·061	0·073	0·319
2	Age (> 30 years)	−12·663	–18·294, –7·033	2·854	−0·311	< 0·001
	Gender (males)	210·683	80·885, 340·480	65·789	0·230	0·002
	Marital status (married)	109·724	4·553, 214·894	53·307	0·150	0·041
	Energy intakes (year)	0·053	–0·069, 0·174	0·062	0·063	0·392
	KAP total^5^	3·097	–1·103, 7·297	2·129	0·107	0·147

¹Urinary sodium excretion as a total for both genders (mg/24 h). ²Reference group for age (20–29·9 years); reference group for gender (female); reference group for marital status (single). ^3^CI. ^4^Standard error. ^5^Total score of food-related knowledge, health-related knowledge, a score of attitudes and a score of practice. ^6^Urinary potassium excretion as a total for both genders (mg/24 h).

A two-stage stepwise multiple regression analysis was conducted (Table 7-B) to assess the association of urinary potassium excretion with KAP after controlling it for the influence of age, gender, marital status and energy intake. Age and gender were significant predictors of urinary potassium excretion (*P* < 0·05). The four variables in step 1 explained 13·3 % (F (4, 185) = 7·109, *P* < 0·001) of the variance in urinary potassium excretion. In step 2, the added KAP total variable did not significantly provide an additional explanation of the variance in the urinary potassium excretion (*P* = 0·147). In the final model, both energy intake (*P* = 0·392) and KAP total (*P* = 0·147) failed to predict urinary potassium excretion, while age (B = –12·663, *P* < 0·001, *r* = –0·311), gender (males) (B = 210·683, *P* < 0·001, *r* = 0·230) and marital status (B = 109·724, *P* < 0·041, *r* = 0·150) were predictors for urinary potassium excretions.

## Discussion

To our knowledge, this is one of the few studies in the UAE to report the 24-h urinary sodium excretion levels using the WHO/PAHO protocol^(12)^ and the KAP questionnaire. However, this is the first study that measured the relationship between urinary sodium execration and food-related knowledge and urinary excretion ratio of sodium to potassium among a group of healthy residents of the UAE.

A 24-h urinary sodium excretion is considered the gold standard method to estimate sodium intake^(27)^ since this period is necessary to capture the marked diurnal variation in sodium, chloride and water excretion. In healthy individuals, electrolyte excretion normally reaches the maximum at or before midday and the minimum at night towards the end of sleep^(28)^. In the present study, several methods were used to confirm the adequacy of 24-h urine collection, including the inclusion criteria recommended by WHO/PAHO protocol, such as urine log sheet and urine volume, as well as the creatinine levels as recommended by the WHO and other studies^(10,12,28–32)^. In addition, the cut-off point for creatinine level was expressed in mg/kg body weight and with a total of 24-h creatinine level as mg/d according to gender^(10,15,31,32)^.

The results of the present study showed a 3·6 % increase in salt consumption in a sample of the UAE population compared with a past study conducted in 2015^(10)^, which showed that the level of salt intake was 6783·5 mg in 2015 compared with 7040·5 mg in the present study. Similar findings on high salt intake were reported in Morocco with an intake of 7·1 g/d^(31)^. These findings highlight the fact that there has been no change or reduction in the salt intake in the UAE population since 2015. A study conducted in Eastern Saudi Arabia presented the mean intake of sodium assessed by a 24-h sodium excretion to be 3·2 ± 1·1 g/d (8·0 g salt/d)^(32)^. In a Jordanian study using 24-h urinary sodium excretion, the intake of salt was doubled (10·4 g/d) to the current WHO recommendation with an average sodium intake of 4·1 g/d. Likewise, a study conducted in Oman using the National Nutrition Survey based on a 24-h dietary recall noted the average intake of salt to be 11–12 g/d^(33)^, which is also significantly higher than the WHO recommendation. Two further studies analysing food consumption in Kuwait^(34,35)^ reported that the average salt intake was within 8–10 g/d. Both studies in Oman and Kuwait recommended the 24-h urinary sodium excretion method to have accurate results of sodium and salt intakes in the population.

Globally, the mean intake of sodium is high in East Asia, Central Asia, Eastern Europe, Central Europe and Middle East/North Africa ranging between 3·9 and 4·2 g/d, which is equivalent to 9·75–10·5 g/d of salt^(36)^. These results exceed the WHO recommendations, which align with our findings. Moreover, the mean urinary sodium excretion in Japan and the UK was 4·47 ± 1·6 g/d and 3·9 ± 1·3 g/d, respectively, which was attributed to high intake of canned and processed foods^(37)^. Moreover, in the present study, the salt intake for males was significantly higher (*P* = 0·003) than for females. This finding is not limited to the present study as similar findings were reported in other studies conducted in the Kingdom of Saudi Arabia and Malaysia^(28,38)^.

Furthermore, several studies conducted in Europe showed the mean sodium intake for males was higher than for females^(39)^, which was confirmed by another study conducted in the adult population in the USA^(40)^. This finding was discussed and explained by two other studies^(41,42)^, which reported that females preferred unsalted foods in the menstrual phase more than in the luteal phase of the cycle. Moreover, statistical analysis revealed significant differences in the preference rating between the menstrual phase and the other two phases (luteal and follicular phases). There was no significant difference in preference between the luteal and follicular phases.

High dietary sodium and low dietary potassium intakes are linked to elevated blood pressure and an increased risk of CVD, which may contribute to the development of NCD^(43)^. In the present study, 24-h urinary potassium excretion was significantly below the WHO-recommended amount of 3·510 g/d^(2)^. This finding aligns with the 2005 UAE Global School-Based Student Health Survey, which reported a high incidence of insufficient fruit and vegetable consumption, a trend that worsened by 2016^(40)^. Another study involving 620 participants from the UAE found that only 28 % met the recommended daily intake of fruits and vegetables, contributing to low potassium intake^(44)^.

The UAE has undergone a rapid transition from a traditional semi-urban lifestyle to a modern urbanised society since the 1960s, following major oil discoveries^(45)^. This shift has led to dietary changes, including frequent snacking, replacing traditional foods with energy-dense fast foods and substituting water with soft drinks, resulting in low fruit and vegetable consumption^(16,45)^.

The 24-h urinary potassium excretion is correlated with dietary potassium intake^(8,28)^. Moreover, the sodium-to-potassium ratio in the present study was 1·15 ± 0·30, thus suggesting that not only did sodium intake for this study exceed the WHO recommendations but also potassium intake was low. A high urinary sodium-to-potassium ratio can be an indicator of the need for reducing sodium intake and increasing potassium intake^(10)^. Therefore, WHO has suggested a sodium-to-potassium ratio of approximately 1·00, which is associated with a low risk for the development of CVD^(3)^.

Furthermore, the macronutrient distribution for carbohydrates, protein and fat was within the Acceptable Macronutrient Distribution Range^(7)^. The mean energy, carbohydrate, protein and fat intakes observed in this study were like those reported in other studies of the UAE population^(18,46)^. However, fibre intake was about 50 % of the adequate intake recommendation, posing a potential risk for NCD in this population^(46)^.

In the present study, 65·3 % of the participants exceeded the WHO recommendations for salt intake, while only 20 % admitted to doing so. This suggests that around 45 % were unaware of their high salt consumption. The study also found that over 60 % of participants had limited knowledge about the main dietary sources of salt. These findings align with a Canadian study where less than 50 % of the participants knew the salt content in various foods like processed cheese and canned vegetables^(47)^, though 70 % could identify health issues related to salt consumption. A significant negative correlation between knowledge, attitudes and practices (and urinary sodium excretion was observed, similar to studies in Northern India^(48)^ and Malaysia^(49)^. This was particularly evident in food-related knowledge, with participants failing to identify low-salt foods. Additionally, participants’ inability to recognise bread and cornflakes as high-sodium foods was comparable to findings from a study among university students in Sharjah, UAE^(18)^.

Cereal products, in particular bread (a popular staple food in the Arab countries), were found to be the major contributor of salt in the Gulf diet^(6)^. Moreover, in Kuwait, a neighbouring Gulf country, it was indicated that bread was the second main source of salt after Kuwaiti composite dishes prepared at home^(6)^. Similarly, in Qatar, the government found that the main source of salt in the Qatari diet was bread and other baked products^(6)^. Low knowledge of sodium/salt contents of foods, particularly bread (Arabic/Iranian), may be considered one of the main reasons behind high salt intake in the UAE. In addition, adding salt to the food during cooking and before tasting is also considered one of the factors that contribute to the high salt intake in the UAE population. This indicates that education and public awareness programmes are required to be established so that the general population is aware of salt portion sizes and the salt content of staple, processed and other foods in general.

A recent systematic review and meta-analysis determining consumer acceptance of reformulated food products showed that salt could be reduced by about 40 % in bread and approximately 70 % in processed meats without significantly affecting consumer acceptability^(50)^, which can be used as an initiative for the UAE to reduce salt intake effectively. A combined approach for salt reduction programmes was used by the UK and South Korea; these approaches focused on improving consumer awareness through campaigns, increased availability of low-sodium foods at school and voluntary reformulation of processed foods. These component initiatives for salt reduction programmes achieved major reductions in the population’s sodium intake (15 % in UK adults and 24 % in South Korean adults)^(9)^.

Moreover, the results of the present study indicated that there is very high sodium with low potassium intakes within the general population of the UAE, which consequently may increase the risk of hypertension, CVD and other NCD. This accelerates the need to initiate salt reduction programmes to reduce the risks of NCD.

Despite our significant findings, a limitation of this study was that urinary sodium was assessed by a single 24-h urine collection, and this may not represent the average sodium intake in a person due to the daily individual variability. However, a single urine measurement is considered a more accurate measure of sodium intake at a population level though possibly less accurate for individuals^(28,31)^. Another limitation was that the food record was self-administered.

### Conclusion

The mean 24-h urinary sodium excretion in the present study exceeded the WHO’s recommended sodium intake and was consistent with findings from reports published in 2015. Additionally, none of the participants met the WHO recommendation for potassium intake, indicating issues with both high sodium and low potassium intake.

Participants’ food and health-related knowledge were classified as poor and fair, respectively. These findings highlight the need for educational and public awareness programmes that focus on food-related knowledge, including salt portion sizes and the salt content of staple foods, processed foods, drinks and other foods. There is also a need for regulations aimed at reformulating food products, particularly staples like bread. National campaigns should emphasise increasing fruit and vegetable intake. Nutrition awareness and educational programmes should be incorporated into school curricula to improve nutritional status and practices among young people.
